# *N*,*N*-chelated nickel catalysts for highly branched polyolefin elastomers: a survey

**DOI:** 10.1098/rsos.180367

**Published:** 2018-07-25

**Authors:** Qaiser Mahmood, Wen-Hua Sun

**Affiliations:** 1 Key Laboratory of Engineering Plastics and Beijing National Laboratory for Molecular Science, Institute of Chemistry, Chinese Academy of Sciences, Beijing 100190, People's Republic of China; 2 CAS Research/Education Center for Excellence in Molecular Sciences, University of Chinese Academy of Sciences, Beijing 100049, People's Republic of China

**Keywords:** thermoplastic elastomer, nickel pre-catalysts, ethylene polymerization, mechanical properties

## Abstract

The physical properties and end applications of polyolefin materials are defined by their chain architectures and topologies. These properties can, in part, be controlled by a judicious choice of the steric and electronic properties of the catalyst and, in particular, the ligand framework. One major achievement in this field is the discovery of thermoplastic polyolefin elastomers that combine the processing and recyclable characteristics of thermoplastics with the flexibility and ductility of elastomers. These polymers are highly sought after as alternative materials to thermoset elastomers. In this perspective, works in the literature related to the development of nickel catalysts as well as their implementations for the synthesis of polyolefin elastomers are summarized in detail. Throughout the perspective, attention has been focused on developing the relationship between catalyst structure and performance, on strategies for the synthesis of polyolefin elastomer using nickel catalysts, on properties of the resultant polyolefin, such as degree of branching and crystallinity, as well as on their effects on mechanical properties. The future perspective regarding the most recent developments in single-step production of polyethylene elastomers will also be presented.

## 1. Introduction

During the last 50 years, polyolefins represent the plastic with the greatest worldwide demand, and indeed, this material continues to experience an ever-growing demand in medical applications and day-to-day commodity materials such as cables, wires, pipes and fibres. One class of polyolefins receiving growing attention is the thermoplastic polyolefin elastomer (TPE). As a promising alternative material to thermoset elastomers, this type of elastomer is gaining immense interest due to its intriguing flexibility and ductility properties that resemble vulcanized rubber, and its processing and recycling that resemble thermoplastic polymer [1–5].

The history of polyolefin polymerization catalysts commenced with Ziegler and Natta's discovery of heterogeneous catalysts [6–8], which have been found to be a workhorse of the polymer industries. Despite the many advantages of these catalysts, the uncertainty as to the nature of the multi-active sites has hindered better understanding of their polymerization mechanism. By contrast, the evolution of well-defined soluble complexes represents a real milestone in understanding the relationship between their catalyst structure and their catalytic performance [9–32]. The precise control over the topologies of polymer has been attained through ligand modification of metallocene catalyst systems [9–13], affording soft, ductile and flexible materials that have uses as elastomeric material in various applications such as in the automotive, electronics, hose and clothing industries [1–5,33,34]. However, materials derived from metallocene catalysts are composed of a statistical copolymer composition which suffers from an imprecise correlation between comonomer percentage and polymer property. For example, increase in the comonomer ratio gives rise to highly ductile material, which limits their high-temperature processing applications [35,36]. To solve this issue [37–48], many researchers from academia and industry have focused on living polymerization to produce block copolymers using a simple olefin monomer for the synthesis of polyolefin elastomers. In living polymerization, polymer chains are grown without termination, enabling the production of block copolymers bearing soft (amorphous) and hard (semi-crystalline) segments using the sequential addition of monomers. Numerous efforts have been made in this approach either using metallocene or non-metallocene early-transition metal catalysts. These catalysts provide vast fundamental knowledge, but the synthesis of precise block copolymers and the time-consuming and inefficient processes make these systems commercially impractical.

Meanwhile, further breakthroughs towards catalytic systems for the synthesis of block copolymers have been presented by Coates & co-workers [49–52]. They developed oscillating catalyst systems that reversibly switch the stereochemistry on the time scale affording polypropylene with both semi-crystalline isotactic and amorphous atactic segments. In contrast with the living polymerization catalysts, these catalysts give multiple chains and generate multi-block-type copolymer. However, the high glass transition temperature (*T*_g_) of atactic blocks limits their use at low-temperature processing (0°C). As a result, polypropylene materials are hard elastomers at room temperature, but become brittle glasses below room temperature [53]. In 2006, scientists at Dow chemical company reported another ingenious approach for the synthesis of multi-block polyolefin elastomers [54]. Using chain-shuttling agents between the two catalyst systems with different modes of polymerization, olefin block copolymers can be produced containing hard blocks of ethylene and soft blocks of α-olefin in a process called chain-shuttling polymerization. These systems can indeed produce multiple polymer chains but suffer from the complexity of the process.

Most of the aforementioned approaches use early-transition metal catalysts for α-olefin polymerization which tend to be poisoned by polar impurities. In 1995, Brookhart and co-workers [55,56] reported α-diimino-Ni(II) and -Pd(II) catalysts for ethylene polymerization. Because of the chain-walking or living polymerization behaviour, these catalysts are extensively used to produce a range of polymeric products from linear through to semi-crystalline to highly amorphous polymers [57–68]. Recently, this strategy has been employed for the synthesis of polyethylene-based elastomeric material using ethylene solely as the monomer. Most importantly, it is a more convenient, simple, single-step and inexpensive approach, and the microstructure can be controlled by just varying the reaction conditions and catalyst to achieve promising mechanical properties similar to commercial polyolefin elastomer (CPOE) [69,70]. Besides being a source of academic interest, the new catalytic systems and their resulting elastomeric products have displayed genuine potential for industrial applications.

This perspective describes all those strategies of α-olefin polymerization in which *N*,*N*-chelated nickel catalysts are employed for the synthesis of TPEs as well as summarizes the development of the ligand framework of these nickel catalysts. Early-transition metal catalysts and approaches that are used for the production of polyolefin elastomers are considered beyond the scope of this perspective and may be found elsewhere [71–76]. Although nickel catalysts have been summarized in previous articles, the focus has been mainly on catalyst structure–activity relationship [28,29,57]. In the review, our effort is to present a new perspective in the development of the nickel-based strategies with particular regard to the mechanical and intrinsic polymer properties resulting from variations in the catalyst structure. At the end, a future outlook of this field will be presented.

## 2. Characteristics of polyolefin elastomers

**Figure 1.**
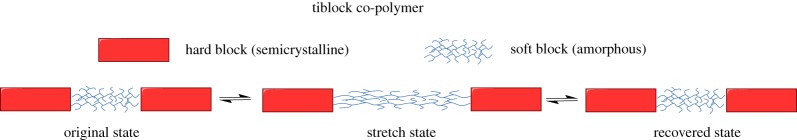
Elastic behaviour of α-olefin-based block copolymers.

TPE is a unique group of polyolefins that has gained high commercial and academic interest because of their characteristically hybrid properties of plastic and elastomers [1–5]. Typically, elastomers such as vulcanized rubber are chemically cross-linked amorphous materials and commonly suffer from reprocessing, recycling and/or reusing problems, which in turn raise the price of the end products [1–5]. As an alternative material for thermoset elastomers, TPEs are highly sought after due to their key advantages over vulcanized rubber. However, simple linear low-density polyethylenes are copolymers based on a statistical mixture of ethylene and α-olefins that show imprecise correlation properties: for example, increasing the comonomer ratio gives rise to highly flexible materials [35,36]. The upshot is that α-olefin-based copolymers have limited use for high-temperature applications. On the other hand, homo-/co-polymerization-based polymers could provide a means to break this long-standing issue of property correlations and extend the scope of polyolefins into more demanding applications. However, synthesis of the block-based copolymer/homopolymer represents the quintessential example of the convergence of strategic design of catalysts and creative techniques leading to an essentially new material. Olefin-based block polymers are composed of semi-crystalline ‘hard’ segments with high melt (*T*_m_) or glass transition temperatures (*T*_g_) and amorphous ‘soft’ segments with low glass transition temperature. Promising elastic properties can be obtained if the microstructure of tri- or multi-block copolymer contains a sequence of two hard segments separated by a soft segment in a linear fashion. Under the applied stress, the hard segments work as physical cross-links and recover the deformations induced by applied stress. The general representation of elastic behaviour of α-olefin-based block copolymers is given in figure 1.

## 3. Types of strategies based on monomer

Considering the low cost of ethylene, propylene and α-olefin monomers, many industrial and commercial efforts have focused on the development of strategic design of catalysts and the methodology to access the α-olefin-based thermoplastic elastomers (TPEs). On the basis of monomer use for the polymerization, these methodologies are classified into α-olefin co-polymerization, propylene polymerization and ethylene polymerization.

### 3.1. Living/chain-walking polymerization

**Scheme 1.**
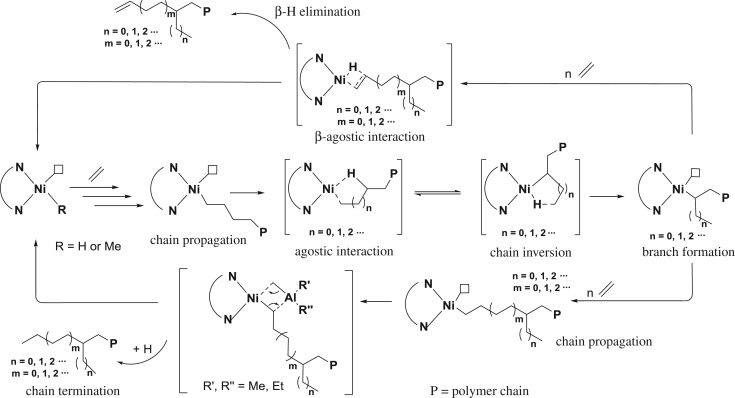
Chain-walking mechanism for ethylene polymerization.

Compared with early-transition metal complex catalysts for α-olefin polymerization, late-transition metal complex catalysts give highly amorphous polymers containing high content of branches. Late-transition metal complex catalysts such as α-diimine-Ni(II) or -Pd(II) complex catalysts through a process called the chain-walking mechanism can generate different lengths of branches ranging from methyl, ethyl, propyl, butyl to long-chain branches along the backbone of the polymer chain, making these polymers highly branched and amorphous in nature. Following the initial insertion of an ethylene monomer, multiple coordination steps lead to the growing polymer chain. During the chain propagation step, *β*, *γ*, *δ *… -agostic hydrogen interactions with the metal centre are likely to take place. Following rotation, reinsertion with opposite geometry and migration of hydride to the terminal carbon generate the methyl, ethyl, propyl or longer branches in the growing polymer chain depending on what type of agostic interactions occur (scheme 1). Since the seminal discovery of α-diimine-Ni(II) catalysts for olefin polymerization, both experimental [77–82] and theoretical [83–89] studies have been performed to gain a deeper insight into the chain-walking mechanism, which underpins the observed branching architectures generating during ethylene and α-olefin polymerizations. Recent studies dictated that polymer properties can be controlled by variation in the reaction parameters such as pressure of ethylene and reaction temperature. More linear polyethylene can be accessed at high ethylene pressure with low reaction temperature.

**Scheme 2.**
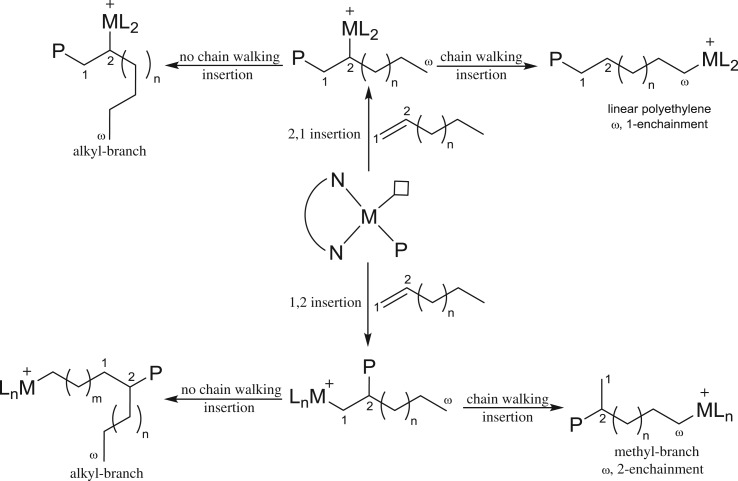
Chain-walking mechanism for α-olefin polymerization.

The polymerization of α-olefins by early-transition metal complexes exhibits selectively 1,2 insertion of monomer; subsequently *ω*,2 enchainment affords poly(α-olefins) with alkyl side chains [90–92]. Sometimes, 2,1 insertion with *ω*,1 enchainment also occurs, which has been known as regio-errors [93]. By contrast, late-transition metal complex catalysts such as α-diimine-Ni(II) and Pd(II) complex catalysts show that the ‘chain-straightening’ phenomenon with 1,2 or 2,1 insertion of the monomer followed by complete chain walking of the catalyst to the *ω*-carbon of the growing polymer chain through *ω*,2 or *ω*,1 enchainment, respectively, leads to the relatively linear polyethylene (scheme 2) [56,94,95]. 2,1 insertion with subsequent *ω*,1 enchainment gives essentially linear polyethylene, whereas 1,2 insertion of monomer with *ω*,2 enchainment affords fewer methyl branches per 1000 carbon atoms. Reaction conditions, namely temperature and monomer concentration, also play a crucial role to control the insertion of monomer and resultant microstructure of polyethylene. The regio-chemistry of monomer insertion is critically important to control the crystallinity of the polymer, which in turn plays a vital role in determining the elastomeric properties of the polymer.

### 3.2. α-Olefins co-polymerization

**Figure 2.**
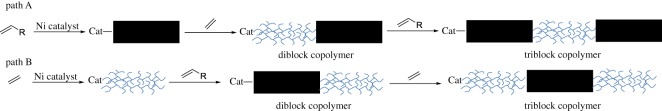
Sequential addition of monomer strategy.

The polymeric materials with desirable physical properties depend on the ability to control the molecular weight and other structural features of the polymer. To date, a plethora of stereo- and regio-selective catalysts have emerged that exhibit living polymerization [55,96–99]. Particular development in this field has been achieved for late-transition metal catalysts in the last two decades [100–102]. Rivalling well-established early-transition metal-based catalysts for α-olefin polymerization, now a number of successful examples of late-transition metal complex catalysts are also available that show high activities, stereo- and regio-selectivity, high precision of polymer and living polymerization behaviour [103]. With precise control of the regio-chemistry of monomer insertion, the nickel complex catalyst can generate TPEs containing polyethylene as an amorphous block and poly(α-olefin) as a semi-crystalline block. A sequential addition of the monomer provides a means to form a sequence of alternative semi-crystalline hard segments and amorphous soft segment copolymer (figure 2) [46,47].

**Figure 3.**
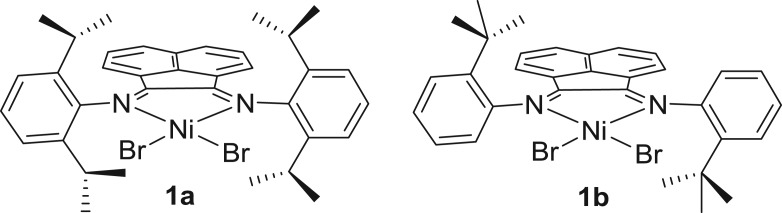
α-Diiminoacenaphthene-nickel pre-catalysts.

In 1996, Brookhart and co-workers [56] for the first time employed α-diimine-nickel (II) halide complexes **1a** and **1b** (figure 3) for a block polyolefin copolymer. At low temperature, the sequential addition of propylene and 1-hexene or 1-octene in the presence of the **1a**/MMAO catalyst system resulted in well-defined high-molecular-weight A–B or A–B–A-type copolymer, whereas the semi-crystalline A block was based on 1-hexene or 1-octene and the amorphous B block was derived from propylene. **1a**/MAO tends to prefer 1,2 insertion of 1-hexene and this tendency decreases with increase of temperature from 0 to 50°C. By contrast, 2,1-insertion was achieved with **1b**/MAO bearing ‘*t*-butyl’ (scheme 2). This might be due to the different regio-chemistry of the ‘*t*-butyl’ group and steric effects. However, these polymers were empirically reported as elastomeric materials, but no further mechanical properties were investigated.

**Figure 4.**
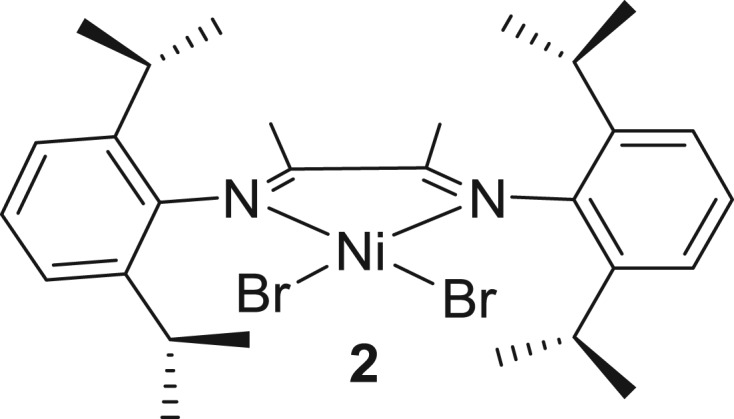
α-Diiminobutene-nickel pre-catalyst.

Ricci and co-workers [104] recently investigated the same approach using catalyst **2** (figure 4) for the synthesis of di- and triblock copolymers from 1-dodecene and ethylene at room temperature. The polymerization of 1-dodecene by the catalyst **2**/MMAO and then the addition of ethylene under 1 atm followed by the formation of a second block of 1-dodecene result in triblock poly(1-dodecen)-polyethylene-poly(1-dodecen) abbreviated as a A–B–A copolymer. Comparatively, the A–B–A triblock copolymer displays better elastomeric properties than a diblock copolymer; maximum values of elongation at break (approx*.* 994%) allied with the highest ultimate tensile strength (18.3 MPa) and a Young's modulus of 28.2 MPa were reported. However, these copolymers displayed poor elastic recovery properties and suffer from permanent deformation after stretching; stress–strain curves of block copolymer exhibited permanent deformation after the first cycle of the stress–strain recovery test. Only 35% of elastic recovery was observed after 10 cycles. Overall, mechanical properties depend on the crystallinity, lengths and composition of the blocks.

Bertini and co-workers [105] have also employed the same nickel catalyst **2** for 1-octene polymerization and studied the mechanical and thermal properties in detail. Complex **2** displays high ToF (888 h^−1^) with good conversion up to 67%. As a general observation, the value of Young's modulus varies from 9 to 2 MPa depending on the crystallinity of the polymer. Notably, the highest ultimate tensile stress (*σ*_b_ = 14 MPa) associated with the strain at break (*ϵ*_b_ = 1337%) was observed for the sample of poly(1-octene) having 58 branches per 1000 carbon atoms, polymer melting temperature (56°C) and high crystallinity (*T*_c_ = 11.5%). However, tensile strength and associated elongation at break are independent of the polymer crystallinity, molecular weight and total number of branches.

**Figure 5.**
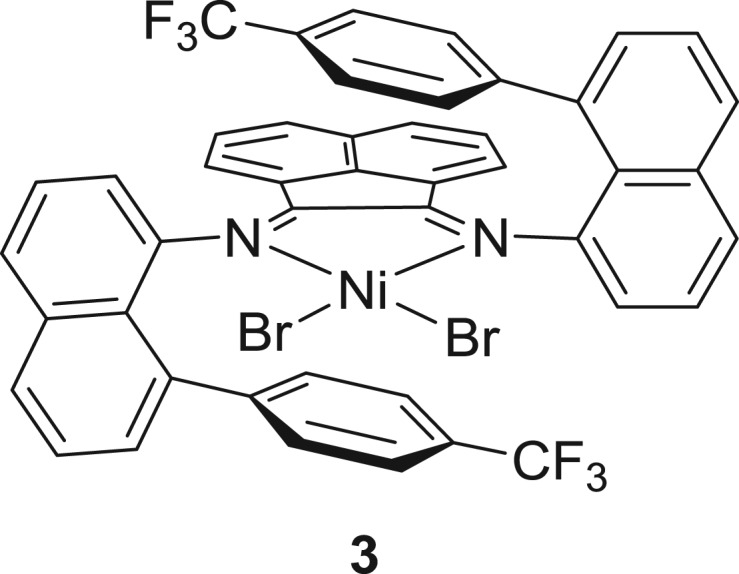
Sandwich-type α-diiminoacenaphthene-nickel pre-catalysts bearing arylnaphthyl groups.

Coates and co-workers recently employed a more advanced type of sequential addition of monomer technique and successfully prepared polyolefin TPEs. In this study, an α-diimine-Ni(II) catalyst bearing arylnaphthyl groups lying above and below the metal centre, the so-called sandwich-type complex **3** (figure 5) [106], was used to generate a block copolymer with an alternative sequence of 1-decene (semi-crystalline) and ethylene (amorphous) segments. Unlike the typical sequential addition technique (figure 2), which normally requires full consumption of the initial α-olefin monomer before the next addition of monomer, ethylene pressurized directly without waiting for full consumption of the first monomer to access the diblock copolymer. With the low concentration of 1-decene and the fast polymerization rate of ethylene, insertion of 1-decene could be negligible. After accessing the soft segment with ethylene monomer of required length, excess ethylene was exchanged with nitrogen and this was followed by the addition of 1-decene monomer, affording a well-defined triblock copolymer. The triblock copolymer shows good mechanical properties such as high elongation at break (maximum *ϵ*_b_ = 750%) with an ultimate tensile strength of 23.3 MPa and improved elastic recovery up to 80–85%, and similar mechanical properties were exhibited by penta- or heptablock copolymers. However, relatively lower elongation at break was seen in the higher block copolymer (*ϵ*_b_ = 570–630%). Surprisingly, diblock and statistical polymer displayed comparable mechanical properties, even higher elongation at break in the range of 780–1120%. The results of these experiments proved that the higher number of block-containing copolymer samples have more ability to resist the deformation when compared with the lower number of block-containing copolymers (diblock, statistical). Furthermore, copolymers with lower number of blocks can elongate before the break point, but the elongation cannot be recoverable due to the absence of or the low number of semi-crystalline segments. Extremely high numbers of hard segments also disfavour the promising elastic recovery. These materials show promising mechanical properties; however, vulcanized rubber is the benchmark as it shows nearly perfect elastic recovery. Further tuning of the catalyst and optimization of reaction conditions have the possibility to improve the chain-walking polymerization in order to increase the number of soft and hard segments, which ultimately advances the mechanical properties and the value of the materials.

### 3.3. Polypropylene-based thermoplastic elastomer

**Figure 6.**
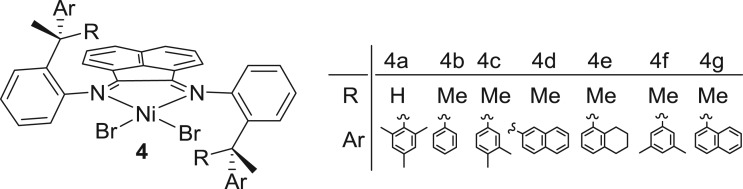
C_2_-symmetric α-diiminoacenaphthene-nickel pre-catalysts.

**Scheme 3.**
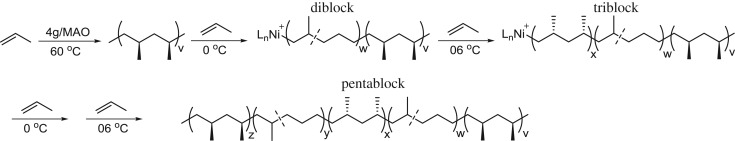
Synthesis of propylene-based tri- and pentablock copolymers.

Propylene is the second simplest α-olefin that contains only one more carbon than the simplest olefin, ethylene. The prochirality of this extra carbon works as a gear to change the physical properties of the polymer ranging from semi-crystalline to amorphous depending on the tacticity of the polymer. Atactic polypropylene is a completely amorphous material having limited industrial usage (e.g. adhesive, caulks and sealants), whereas syndiotactic and isotactic are semi-crystalline in nature, showing relatively high melting temperatures of 150°C and 165°C, respectively. There are numerous catalysts including homo- and heterogeneous catalysts that are capable of atactic and isotactic propylene polymerization. Coates and co-workers [107] have employed C_2_-symmetric σ-diimine-nickel complex **4** (figure 6) for propylene polymerization. Upon treatment with methylaluminoxane (MAO), the catalysts are highly isoselective at low temperature (−60°C) and give highly regio-regular isotactic polypropylene through 1,2 enchainment, whereas at higher temperature (0°C) provide regio-irregular polypropylene composed of 1,2- and 3,1-enchainment (scheme 3). Using temperature-dependent regio-selectivity, different blocky polypropylenes composed of alternative sequences of isotactic semi-crystalline and regio-irregular segments were prepared, and their mechanical properties have been studied. At 20°C, the pentablock polymer sample obtained by **4a**/MAO displayed the highest elongation at break of 2200% allied with the highest stress of 245 MPa. The second best value of elongation at break is 1300% associated with an ultimate stress of 88 MPa. At 65°C, most of the polypropylene sample exhibited better mechanical properties than those that were measured at lower temperature. An increasingly isoselective-based hard block displays higher melting temperature and heat of fusion, which probably favour good mechanical properties at high temperature.

### 3.4. Polyethylene-based thermoplastic elastomer

Physical properties of polyethylene and their applications highly depend on the microstructure and topology, which can be tuned dramatically for specific applications using the different polymerization conditions. The chain-walking mechanism was first reported by Fink and co-workers [58,59], which was later dictated by Brookhart and other researchers in the current form (scheme 1) [60,61]. Under chain-walking ethylene polymerization, polyethylene can be furnished with a high number of branches including methyl, ethyl and propyl depending on the reaction conditions and ligand frameworks. By contrast, the chain-straightening phenomenon gives linear polyethylene (scheme 2). β-Hydride elimination and reinsertion with the opposite regio-chemistry are the main causes to induce the branching content and control the topology of the end polymeric materials. Recently, this strategy has been employed for the synthesis of polyethylene as a thermoplastic elastomeric material. The following sections will summarize the nickel complex catalysts that afford highly branched polyethylene.

#### 3.4.1. α-Diimine-nickel pre-catalysts

**Figure 7.**
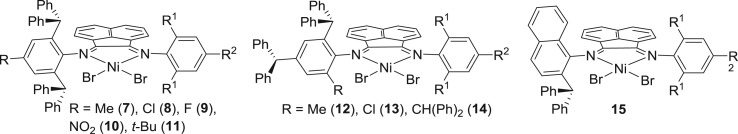
Benzhydryl-containing unsymmetrical α-diiminoacenaphthene-nickel pre-catalysts **7–15**.

Recently, our group has exploited a series of unsymmetrical α-diiminonickel pre-catalysts bearing a benzhydryl group [108]. These complex catalysts showed outstanding catalytic activities and improved thermal stability. More interesting is the microstructural properties of resultant polyethylene such as the high degree of branches, high to ultra-high molecular weight and narrow molecular-weight distributions. Tailoring the ligand structure with suitable substituents and varying the reaction conditions offered a significant control over the number of branches. In 2011, the first example of unsymmetrical 1,2-bis(imino)acenaphthene-nickel(II) halide complexes bearing benzhydryl groups was investigated for ethylene polymerization. Variation in polyethylene properties as a function of these catalyst structures are summarized in table 1. On activation with either MAO or Et_2_AlCl, catalyst **7** [108] (figure 7 and table 1) gives outstanding activities (up to 1.12 × 10^7^ g (PE) mol^−1^(Ni) h^−1^) and produces high molecular-weight polyethylene with a narrow molecular-weight distribution. The random microstructure of the polymer contained high numbers of branches ranging from 79 to 114 branches per 1000 carbon atoms which can be controlled by the optimization of reaction conditions. Stress–strain curves highly depend on the number of branches and crystallinity of the polyethylene [116]. The polyethylene sample with 100 branches per 1000 carbon atoms with crystallinity in the range of 10–13% displayed the best mechanical properties: the ultimate tensile stress and elongation at break are found to be 4.0 MPa and 2125%, respectively. The highest elastic recovery was observed up to 75% with 300% strain after 10 cycles at 45°C. Despite the high elongation at break, it is still necessary to increase the ultimate tensile strength along with ultimate elongation at break. When R belong to the Cl group, complex **8** (figure 7) displayed exceptionally high activities (up to 1.10 × 10^7^ g (PE) mol^−1^(Ni) h^−1^) [109], similar to the activity that was reported using complex **7** [108]. What is more exceptional is the ultra-high molecular weight in the range of 10^6^ g mol^−1^ and the high number of branches, as high as 98 per 1000 carbon atoms at 60°C. With further increases in the negative electronic effect of the R group and the incorporated F substituent, catalytic performance of complex **9** (figure 7) slightly improved to 1.27 × 10^7^ g (PE) mol^−1^(Ni) h^−1^ and produced polyethylene with ultra-high molecular weight [110,117,118]. Computational studies on late-transition metal complexes for ethylene polymerization also justify these values of high activities due to the negative electronic effect of substituents and their ultimate effect on the net charge of the active species [119]. A high number of branches (132 branches per 1000 carbon atoms at 30°C) and narrow molecular-weight distributions are features of the resultant polyethylene. On the other hand, when *ortho*-R is NO_2_, complex **10** (figure 7) exhibited a negative effect on the activity and provided lower productivity of 4.50 × 10^6^ g (PE) mol^−1^(Ni) h^−1^ at 20°C as well as a lower number of branches (up to 50 per 1000 carbon atoms) [111]. Conversely, the NO_2_ group exerted a positive effect on the molecular weight of the resultant polyethylene, showing ultra-high molecular weight up to 3.28 × 10^6^ g (PE) mol^−1^(Ni) h^−1^.

**Table 1.** Variation in polyethylene properties as a function of the non-fluorinated catalyst structure.^a^

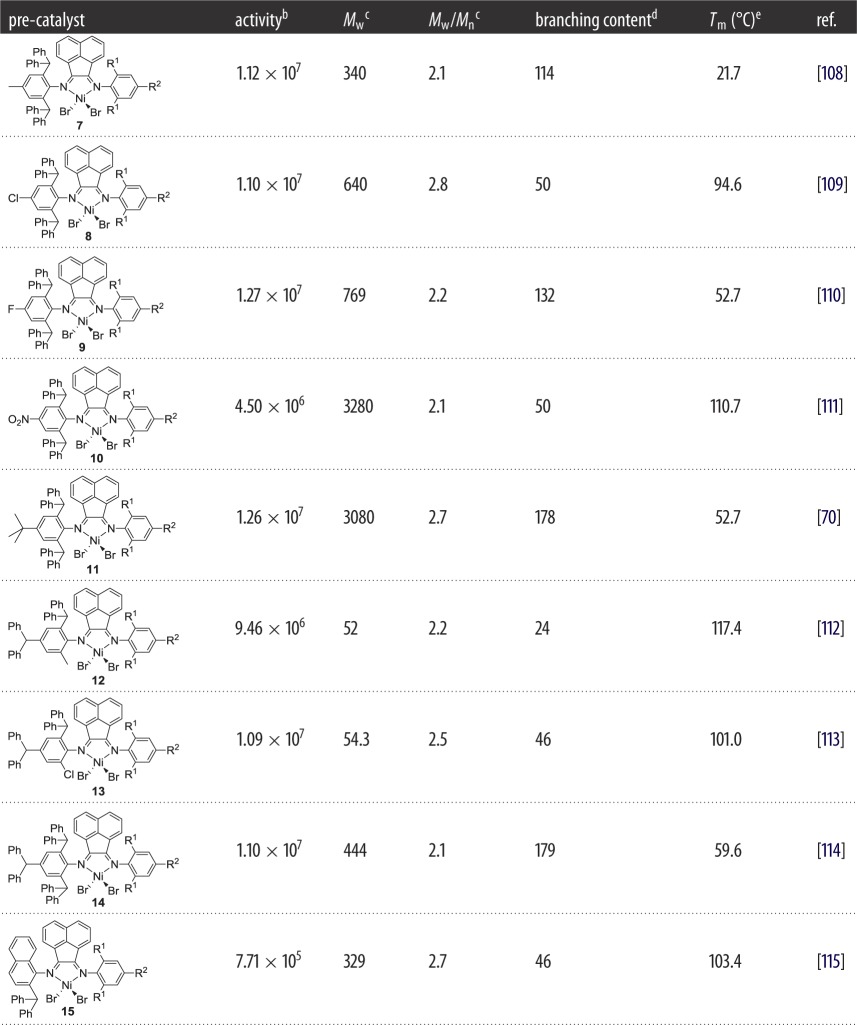

^a^Ethylene pressure 10 atm, toluene as solvent, run time 30 min, R^1^ = Me, Et and ^*i*^Pr and R^2^ = H, Me.

^b^Grams of PE (mol of Ni)^−1^ h^−1^.

^c^Determined by GPC, *M*_w_: kg mol^−1^.

^d^Determined by ^13^C NMR spectroscopy and is the number of branches per 1000 carbon atoms.

^e^Determined by DSC.

To exploit the cause of elastic behaviour of polyethylene which is either due to the branching content and crystallinity or the molecular weight, our group incorporated the *t*-Bu group into the α-diiminonickel pre-catalysts [70]. On treatment with either MMAO or Et_2_AlCl, **11** (figure 7 and table 1) was a highly active catalyst in ethylene polymerization (as high as 1.26 × 10^7^ g (PE) mol^−1^ (Ni) h^−1^) affording hyper-branched polyethylene. Exceptionally high molecular weight in the range of 10^6^ g mol^−1^ (as high as up 3.1 × 10^6^ g mol^−1^) was achieved. Mechanical properties were conducted for high and ultra-high molecular-weight polyethylene, and comparative results indicated that the molecular weight also was somewhat responsible in influencing the mechanical properties. For example, in comparison with high molecular-weight polyethylene (*M*_w_ = 6.4 × 10^5 ^g mol^−1^, branches = 173/1000°C, *X*_c_ = 8.1%), the ultimate tensile strength significantly increased from 6.88 MPa to as high as 13.22 MPa for the sample of ultra-high molecular-weight polyethylene (3.1 × 10^6^ g mol^−1^, branches = 178/1000°C, *X*_c_ = 10.7%), with the associated elongation at break increasing from 630 to 843.9%. The property of ultra-high molecular weight with a suitable number of branches is more influential than only the number of branches or crystallinity to obtain the promising tensile strength, elastic recovery as well as high elongation at break. In comparison with the CPOE [120,121], the sample of ultra-high molecular-weight polyethylene exhibited a value of ultimate tensile strength of 13.22 MPa, similar to the 13.62 MPa for CPOE, while its elongation break, 843.9%, is also similar (*ϵ*_b_ = 845%). These values highlight that these materials offer a promising potential to compete with commercial TPEs. On setting the selected properties of different polyolefins, it is found that polyethylene obtained by complex **11** showed comparable mechanical properties with commercial polyolefin elastomers (CPOE) and higher than those of low-density polyethylene (LDPE), linear low-density polyethylene (LLDPE) and propylene (PP). The selected data such as melting point, molecular weight, crystallinity, tensile strength and elongation at break for various elastomeric materials are presented in table 2.

**Table 2.** Selected data for various types of polyolefins.

polymer type	*T*_m_ (°C)	*X*_c_ (%)	*M*_w_	stress (MPa)	strain (%)	ref.
PE (using **11**, table 1)	53.4	10.7	30.8	13.22	843.9	[70]
CPOE	80.7	14	1.15	13.62	845	[120]
LLDPE	120–125	40–60	—	30	500	[121]
LDPE	106–120	65–80	—	12–15	600	[121]
PP	90–130	10–16	—	16–25	400–800	[13]
natural rubber	—	—	—	13–17	757–787	[5]
polybutadiene (hot GRS)	—	—	—	1.2–1.4	290–815	[5]
*cis*-1,4-polyisoprene	−73^a^	—	—	29	660–850	[3]
butyl rubber	−75 to 67^a^	—	—	17	700–950	[3]

^a^*T*_g_ (°C).

In the follow-up paper, the substitution pattern of the benzhydryl group in the 1,2-bis(imino)acenaphthene-nickel(II) complex has been modified at the 2,4 and 2,4,6 positions affording **12–14** (figure 7 and table 1) [112–114]. High activities with good thermal stability are the prominent features of these complexes. When *ortho*-R is Me, complex **12** (figure 7) in conjunction with MAO exhibited a high activity of 9.46 × 10^6^ g (PE) mol^−1^(Ni) h^−1^ and produced comparatively lower molecular-weight polyethylene [112]. Polyethylene with 24 and 116 branches per 1000 carbon atoms was obtained at 20°C and 80°C, respectively. When *ortho*-R belongs to the Cl group, again exceptionally high activity of 1.09 × 10^7^ g (PE) mol^−1^(Ni) h^−1^ was displayed by complex **13**/EASC (figure 7) [122]. Most importantly, these complexes displayed high thermal stability: maintained a high activity of 3.68 × 10^6^ g (PE) mol^−1^(Ni) h^−1^ at a temperature of 80°C. Regarding the microstructure, the polyethylene produced at 50°C possessed 46 branches, which slightly increased to 59 per 1000 carbon atoms at 60°C. Furthermore, longer branch chains were formed at higher temperature, being attributed to the higher potential of chain termination and migration when compared with chain propagation [123,124]. In exploiting the mechanical properties of homopolyethylene, we employed a set of unsymmetrical 1,2-bis(imino)acenaphthene-nickel(II) complexes bearing a 2,4,6-benzhydryl group substitution pattern (**14**) for ethylene polymerization [114]. On treatment with relatively low amounts of Et_2_AlCl or Me_2_AlCl (200–700 equivalents), complex **14** (figure 7) displayed remarkably high activities for ethylene polymerization (up to 1.1 × 10^7^ g (PE) mol^−1^ (Ni) h^−1^) as well as high thermal stability (approx. 2.97 × 10^6^ g (PE) mol^−1^ (Ni) h^−1^ at 90°C). More notable is the high number of branches varying in the range of 106–179 per 1000 carbon atoms. Regarding the mechanical properties, high ultimate tensile stress (up to 13.52 MPa) with strain at break (218.3%) was observed for the polyethylene sample prepared at 20°C (polymer properties: branches = 106/1000°C, *X*_c_ = 20.7%, *M*_w_ = 4.77 × 10^5^ g mol^−1^, *T*_m_ = 96.9°C). The sample prepared at 80°C showed more promising mechanical properties: 6.25 MPa of ultimate tensile strength with 518.9% strain at break (polymer properties: branches = 145/1000°C, *X*_c_ = 12.9%, *M*_w_ = 3.74 × 10^5^ g mol^−1^, *T*_m_ = 55.6°C). Probably, the highly branched microstructure of these polyethylenes tends to have high physical cross-links which in turn show good tear resistance. Furthermore, these polyethylene samples exhibit high elastic recovery up to 84%. Results of the mechanical measurements claim that the tensile properties of the polyethylene samples were mainly influenced by the branching content and the crystallinity rather than the molecular weight of the polyethylene. In the presence of MAO or Et_2_AlCl co-catalysts, complex **15** (figure 7 and table 1) [115] bearing benzhydryl groups on a naphthyl moiety exhibited moderate activities for ethylene polymerization and the resultant polyethylene showed high molecular weight and a lower degree of branching content (46 branches per 1000 carbon atoms).

**Figure 8.**
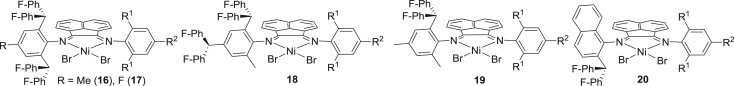
Difluorobenzhydryl-containing unsymmetrical α-diiminoacenaphthene-nickel pre-catalysts **16–20.**

Based on the positive effect of fluoride groups on thermal stability, our group reported the second generation of unsymmetrical α-diiminonickel complex pre-catalysts in which fluorinated benzhydryl groups were incorporated, and their catalytic performance is described in table 2. When *para*-R is Me, complex **16** (figure 8 and table 3) [125] behaved as a high thermo-stable polymerization catalyst with high activities in the range of 10^7^ g (PE) mol^−1^(Ni) h^−1^. Even at 80°C (commonly industrial operating temperature is 80–100°C) a good activity of 4.87 × 10^6^ g (PE) mol^−1^(Ni) h^−1^ was maintained. The more remarkable advantage is that the produced polyethylene possessed high molecular weight with a high number of branches up to 140 per 1000 carbon atoms, which makes it a good candidate for the TPE. On activation with an EASC (relatively lower loading of co-catalyst), complex **17** (figure 8 and table 3) [69] exhibited an extremely high activity of 2.20 × 10^7^ g (PE) mol^−1^(Ni) h^−1^ at 30°C. Improvement in catalytic performance was attributed to the negative electronic effect of the fluorides, and their electronic influences on the net charge of the metal centre are participating. A high number of branches up to 116 per 1000 carbon atoms (methyl (52.6%), ethyl (6.4%) and longer chains (41.0%)), moderate melting points and narrow molecular-weight distributions are the characteristics of the resultant polyethylene. On reducing the steric hindrance near to the active species, unique and quite surprising results were achieved with complex **18** (figure 8 and table 3) [126], such as high catalytic activities (up to 8.52 × 10^6^ g (PE) mol^−1^(Ni) h^−1^) and remarkable thermal stability (with activity of 1.12 × 10^6^ g (PE) mol^−1^(Ni) h^−1^ at 100°C), while producing high molecular-weight polyethylenes (up to 10^5^ g mol^−1^) at elevated polymerization temperature. Furthermore, the resultant polyethylene is highly branched with the branching number ranging from 38 to 100 per 1000 carbon atoms: variation in the total number and type of branches depends on the polymerization temperature. On treatment with either EASC or MAO co-catalysts, complex **19** (figure 8 and table 3) [127] displayed high activities up to 10^6^ g (PE) mol^−1^(Ni) h^−1^ as well as high thermal stability in the range of 60–80°C, producing high molecular-weight polyethylene (*M*_W_ = up to 10^6^ g mol^−1^). The resultant polyethylene possessed a comparatively lower number of branches up to 27 per 1000 carbon atoms. For further exploring the fluoride effect, we incorporated fluorinated benzhydryl to the *N*-naphthyl unit and prepared nickel complex **20** (figure 8 and table 3) [128]. On activation with the EASC co-catalyst, complex **20** (figure 8 and table 3) was a highly productive catalyst for ethylene polymerization and produced high molecular-weight polyethylene with narrow molecular-weight distributions (*M*_w_/*M*_n_ = 1.22–1.99).

**Table 3.** Variation in polyethylene properties as a function of the fluorinated catalyst structure.^a^

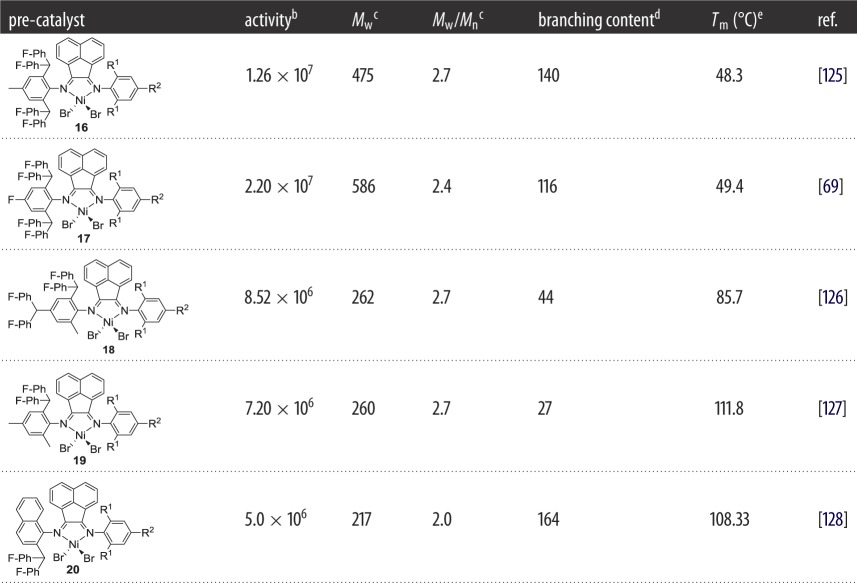

^a^Ethylene pressure 10 atm, toluene as solvent, run time 30 min, R^1^ = Me, Et and ^*i*^Pr and R^2^ = H, Me.

^b^Grams of PE (mol of Ni)^−1^ h^−1^.

^c^Determined by GPC, *M*_w_: kg mol^−1^.

^d^Determined by ^13^C NMR spectroscopy and is the number of branches per 1000 carbon atoms.

^e^Determined by DSC.

**Figure 9.**
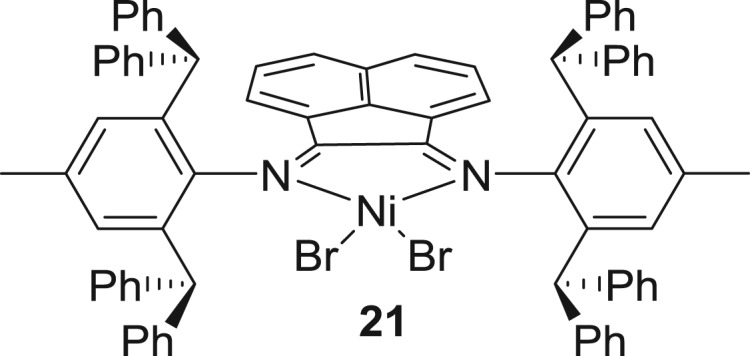
2,6-Dibenzhydryl-substituted symmetrical α-diimine-nickel pre-catalysts, **21.**

Long and co-workers [129] synthesized symmetrical complex **21** (figure 9) that maintains stable turnover frequencies that linearly increase with increase of time, and even show high activity at 100°C. The polyethylene exhibited a moderate number of branches (63–75 branches per 1000 carbon atoms), high molecular weight (*M*_n _> 600 000 g mol^−1^) and narrow molecular-weight distributions (*M*_w_/*M*_n_ ≤ 1.22) up to 100°C. Despite complex **21** showing slightly lower activity than complex **7**, the introduction of dibenzhydryl groups at both *N*-aryl substituents considerably enhanced the thermal stability of the resultant nickel complexes, thus showing high activity.

**Figure 10.**
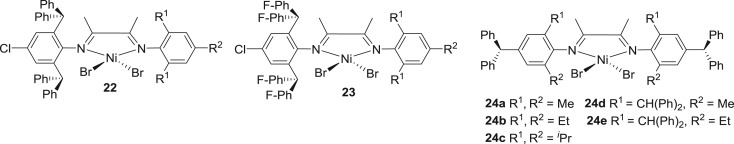
Fluorinated and non-fluorinated benzhydryl-containing unsymmetrical and symmetrical α-diimine-nickel pre-catalysts **22–24.**

Meanwhile, our group has made sequential progress to improve the thermal stability of α-diimine-based nickel complexes. By incorporating the fluorinated and non-fluorinated benzhydryl groups as bulky substituents on the *N*-aryl groups, unsymmetrical complexes **22** (figure 10) [130] and **23** (figure 10) [131] and symmetrical complex **24** (figure 10) [132] have been synthesized in high yields. Complex **22** (figure 10) [130] exhibits high activity, as high as 4.59 × 10^6^ g (PE) mol^−1^(Ni) h^−1^ for ethylene polymerization, and the obtained polymer shows high molecular weights in the range of 10^6^ g mol^−1^ with a high number of branches up to 202 per 1000 carbon atoms. Fluorinated benzhydryl groups containing α-diimine-nickel complex **23** (figure 10) [131] showed slightly higher activity than that found for non-flourinated complex **22** (figure 10) [130] and afforded high molecular-weight polyethylene (up to 1.07 × 10^6^ g mol^−1^) with a high number of branches (up to 220 per 1000 carbon atoms) and bimodal molecular-weight distributions. Upon activation with Et_2_AlCl, complex **24** (figure 10) [132] exhibited slightly lower activity (2.41 × 10^6^ g (PE) mol^−1^ (Ni) h^−1^) generating high molecular-weight polyethylene with a drastically lower number of branches (up to 18 per 1000 carbon atoms) and narrow molecular-weight distributions (PDI = 2–4).

**Figure 11.**
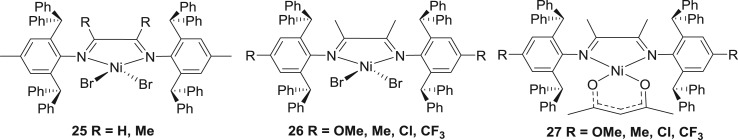
2,6-Dibenzhydryl-substituted symmetrical α-diiminobutene-nickel pre-catalyst **25–27.**

Long and co-workers [133] reported benzhydryl-modified symmetrical α-diimines-nickel **25** (figure 11) for ethylene polymerization. One obvious feature of these complexes is that they exhibit excellent thermal stability: high catalytic activities were maintained at elevated temperature in the range of 80–100°C. Even at 100°C, a high activity of 2.12 × 10^6^ g (PE) mol^−1^ (Ni) h^−1^ was achieved. Regarding the properties of the resultant polyethylene, high molecular weight (*M*_n_ ≤ 6.25 × 10^5^ g mol^−1^) with narrow molecular-weight distributions (*M*_w_/*M*_n_ < 1.31) and a moderate number of branches (63–75 branches per 1000 carbon atoms) are the characteristics of the polymer.

The Chen group reported a series of symmetrical α-diimine-nickel complexes bearing *ortho*-benzhydryl and *para*-substituted electron-donating and -withdrawing groups. These nickel catalysts **26** and **27** (figure 11) [134] are highly active towards ethylene polymerization (up to 6.0 × 10^6^ g (PE) mol^−1^(Ni) h^−1^), but give exceptionally high thermal stability (stable up to 100°C), producing polyethylene with high molecular weight (*M*_n_ ≈ 1.6 × 10^6^ g mol^−1^) and narrow molecular-weightdistributions.

**Figure 12.**
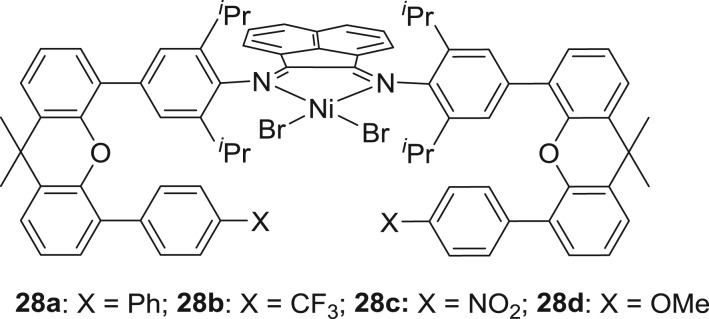
α-Diiminoacenaphthene-nickel pre-catalysts bearing different substituents at remote positions.

Recently, Chen and co-workers [135] reported a series of symmetrical α-diimine-nickel catalysts **28a–d** (figure 12) bearing different substitutions ranging from electron-donating to the electron-withdrawing groups at remote position. In the presence of MAO, nickel complexes **28a–c** (X = Ph, CF_3_, NO_2_) displayed activities in the range of 1.80–3.10 × 10^6^ g (PE) mol^−1^ (Ni) h^−1^) but lower than those of complex **28d** (activity ≤6.94 × 10^6^ g (PE) mol^−1^ (Ni) h^−1^). This higher activity might be due to the interaction of OMe with the active centre. Interestingly, nickel complexes bearing electron-withdrawing groups (CF_3_ or NO_2_) produced a comparatively lower number of branches (48 and 21 per 1000 carbon atoms, respectively) when compared with the Ph substituent counterpart (78 per 1000 carbon atoms). By changing the reaction temperature from 20 to 80°C, the molecular weight of the resulting polyethylene decreased. The measurement of mechanical tests of polyethylene samples prepared under different reaction conditions showed that molecular weights and branching densities are particularly important in inducing the elastic properties. The values of ultimate tensile strength and elongation at break were varied in the range of 3–28 MPa and 300–1800%, respectively. The samples of polyethylene prepared at a lower temperature of 20°C show good ultimate tensile strength, whereas the values of elongation at break are found comparatively lower than the sample prepared at a temperature of 80°C. Meanwhile, the polyethylene samples with lower molecular weight and a high number of branches are more ductile, showing high elastic recovery, as high as 84%.

Pellecchia and his co-workers revisited the classical Brookhart's nickel catalyst **1a** (figure 3) for ethylene polymerization and reported the mechanical properties of the resultant elastomeric materials [113]. The nature of the aluminoxane co-catalyst employed had a marked effect on the number of branches of the resultant polymeric material, which directly influenced the mechanical properties. High ductility and flexibility (elongation at break higher than 2000% with an ultimate tensile strength close to 0.4 MPa) was shown by the highly amorphous polyethylene sample having 80 branches per 1000 carbon atoms. A lower number of branches resulted in the drop of elongation at break up to 1500%, while the ultimate tensile strength was improved to 1.3–1.5 MPa. Further decreases in the number of branches to 49 per 1000 carbon atoms led to further increase in the ultimate tensile strength (*σ*_b_ ≈ 2.5 MPa), but elongation at break again was reduced to half (*ε*_b_ ≈ 1000%). A reduction in branch concentration is advantageous to improve the ultimate tensile strength, but it also reduces the elongation at break. Tuning of the ligand framework, optimizing the reaction conditions and use of appropriate co-catalysts are the parameters that could be beneficial to control the topology of the polyethylene in order to obtain the promising elastomeric polyethylene.

#### 3.4.2. Pyridinylimine-nickel pre-catalysts

α-Diimine-nickel catalyst-based polyethylene as described above have high potential to be used as TPEs because of their unique microstructural properties such as high branching content and high molecular weight of the polymer. In recent years, the attention of researchers has been focused on *N*,*N*-bidentate pyridinylimine-nickel catalysts, as these catalysts exhibit high activities and generate highly branched polyethylene. Hence, extensive efforts have been made to prepare pyridinylimine-nickel catalysts and catalytic behaviour towards ethylene polymerization.

**Figure 13.**
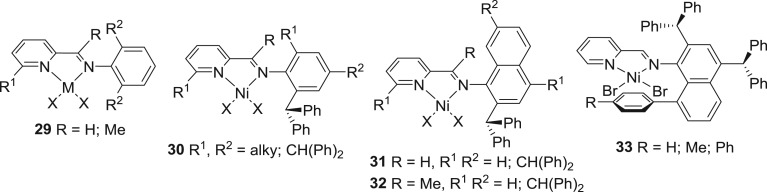
Pyridinylimine-nickel pre-catalysts, **29–33.**

Laine *et al.* [136–138] reported an early example of pyridinylimine-based dimeric nickel complexes **29** (figure 13). In the presence of MAO, complex **29** exhibited a moderate to good activity as high as 6.8 × 10^6^ g (PE) mol^−1^ (Ni) h^−1^ generating polyethylene with a high number of branches (up to 78 branches per 1000 carbon atoms at 40°C) but low molecular weights.

Our group incorporated a benzhydryl group to the pyridinylimine-based nickel pre-catalysts to mitigate the chain transfer reactions and protect the active species in the form of complex **30** (figure 13) [139]. On activation with either MAO or MMAO, significantly high activities were achieved (up to 10^7^ g (PE) mol^−1^ (Ni) h^−1^). A high number of branches and narrow molecular-weight distributions are characteristic properties of the resultant polyethylene. These convincing results motivated us to further exploit the steric effect on the catalysts' properties. In this context, we incorporated the benzhydryl group to the naphthalene-1-amine and reported nickel complex **31** (figure 13) [140]. An exceptionally high activity of 2.02 × 10^7^ g (PE) mol^−1^ (Ni) h^−1^ for ethylene polymerization was achieved. The resultant polyethylene displayed a low molecular weight, narrow distribution and a high number of branches (182 branches per 1000 carbon atoms). Similarly, when R is Me, complex **32** (figure 13) [141] exhibited a slightly lower activity of 1.22 × 10^7^ g (PE) mol^−1^ (Ni) h^−1^ than that reported for complex **31** (figure 13) [140].

Recently, the Chen group employed complex **33** (figure 13) [67] for ethylene polymerization and obtained high activity in the range of 10^6^ g (PE) mol^−1^ (Ni) h^−1^. More interestingly, the catalytic performance of these catalysts gives rise to their high thermal stability, producing semi-crystalline polyethylene with ultra-high molecular weight (up to 10^6^ g mol^−1^).

**Figure 14.**
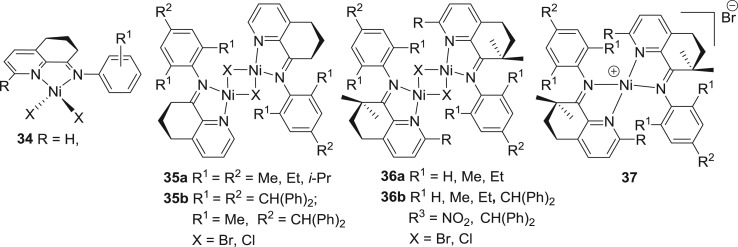
8-Arylimino-5,6,7-tetrahydroquinoline-nickel pre-catalysts, **34–37.**

In the recent years, our group has exploited how the strength of metal–N_imine_ bond in pyridinylimine-nickel complexes influences the catalytic activities of the resultant catalysts. For this, a new generation of pyridinylimine-nickel catalysts are employed for ethylene polymerization in which cycloalkyl groups of different ring sizes are incorporated (5, 6, 7 and 8 carbon-membered rings). In the first instance, 6 membered rings were introduced and thus were prepared nickel complexes **34–37** (figure 14) [142–146]. When R is H, the nickel complex **34** (figure 14) [142] gave good activity for ethylene polymerization and resultant polyethylene showed lower molecular weight with a high degree of branching and narrow molecular-weight distributions. Similarly, halide-bridged binuclear nickel complexes **35a** and **35b** (figure 14) [143,144] were exploited for ethylene polymerization. Complex **36a** is a remarkably efficient catalyst showing activity up to 1.1 × 10^6^ g (PE) mol^−1^ (Ni) h^−1^, and the resultant polymer is waxy in nature, having a high degree of branching content and narrow molecular-weight distributions. Complex **35b** bearing benzhydryl *N*-aryl groups also resulted in high activity (maximum activity: 56.6 × 10^6^ g (PE) mol^−1^ (Ni) h^−1^) for ethylene polymerization and the polymer shows similar properties as seen for those polymers obtained with **35a**. In the second generation of these fused pyridinylimine-nickel catalysts, imine–enamine tautomerization was inhibited by adding *gem*-methyl groups, and complexes **36** and **37** were prepared (figure 14) [145,146]. Upon treatment with either MAO or EASC, these complexes are found to be efficient catalysts for ethylene polymerization and give high activities (up to 64.8 × 10^4^ g (PE) mol^−1^ (Ni) h^−1^). Interestingly, the molecular weight of the resultant polyethylene falls in the range of lower molecular weight but with very narrow molecular-weight distributions (PDI = 1.7–2.0). Terminal unsaturation is also a feature of the resultant polyethylene.

**Figure 15.**
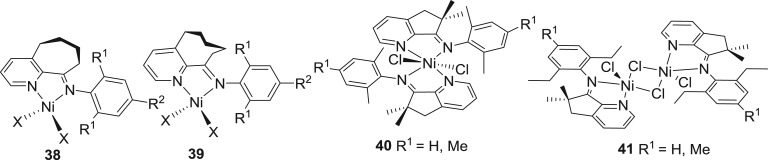
Cycloalkyl-fused-pyridinylimine-nickel pre-catalysts, **38–41.**

Further impact of the fused cycloalkyl on the properties of the nickel complexes was exploited by the expansion or contraction of the fused cycloalkyl groups. The nickel complex **38** (figure 15) [147,148] chelated with the 9-aryliminocycloheptapyridine *N*′*N* ligand displayed comparatively higher activity (up to 6.27 × 10^6^ g (PE) mol^−1^ (Ni) h^−1^) than complex **34** (figure 14) [142] with regard to ethylene polymerization. The obtained polymers are waxes with molecular weight in the range of 1.8 and 6.8 kg mol^−1^, and their molecular weight distribution varies between 1.4 and 1.8. High-temperature ^13^C NMR spectroscopy showed that the polyethylene sample was highly branched polyethylene wax and the branching degree was more than 50 per 1000 carbon atoms. Meanwhile, complex **39** (figure 15) [149] chelated by the 10-aryliminocyclooctapyridine *N*′*N* ligand was prepared. On treatment with MAO or Et_2_AlCl co-catalysts, complex **39** (figure 15) [149] gave high activities in the range of 10^6^ g (PE) mol^−1^ (Ni) h^−1^ and produced polyethylene waxes with lower molecular weight (1.4–6.7 kg mol^−1^) and narrow molecular-weight distributions (PDI = 1.7–2.4), highlighting a single-site catalytic active species. Similarly, our group reported highly strained imino-cyclopenta[b]pyridines for the synthesis of nickel complexes. Towards ethylene polymerization, the active species formed from **40** and **41** (figure 15) [150] can be formed efficiently (3096–5478 h^−1^ at 20°C) affording highly active catalysts (5.0 × 10^6^ g (PE) mol^−1^ (Ni) h^−1^). Detailed microstructural analysis revealed the presence of vinyl and vinylene unsaturation with a ratio of vinyl : vinylene = 22 : 1.2 at 60°C.

## 4. Conclusion and future outlook

In this perspective, different strategies such as α-olefin co-polymerization, propylene polymerization and ethylene homo-polymerization have been summarized for the synthesis of TPEs, all of which make use of *N*,*N*-chelated nickel catalysts. The ethylene homo-polymerization approach can be considered a cost-effective alternative to more complicated and multi-step approaches to make elastomeric materials, as it requires only a single step for the polymerization. However, some issues need to be addressed before employing this approach for any practical applications. For example, the molecular weight of the polymers is not sufficiently high and the elastic properties are not suitable below room temperature. A further key issue is the low thermal stability of these nickel catalysts, which substantially affects not only the catalytic activities but also the mechanical properties of the resultant polyethylene. As with temperature instability issues, the need to control the mechanical properties at elevated temperature is also a key issue. A relationship between structural variations in the catalyst and their catalytic performance showed that highly bulky 1,2-bis(imino)acenaphthene-nickel(II) complexes are particularly versatile candidates as these catalysts allow for the fine-tuning of their ligand structure so as to control the catalytic performance and properties of the polymer [151].

We believe that there is tremendous potential for further ligand design in this area. The introduction of benzhydryl groups at the ortho positions of the *N*-aryl groups and their impact on polymer structure and thermal stability highlights the considerable opportunities for tuning the ligand backbone. However, despite the relative infancy of this field, the properties of these homo-polymerized elastomers already show properties reminiscent of CPOEs. Looking beyond the molecular level, improvements likely to be made through systematic variation in the temperature and pressure offer a physical means to further optimize the elastomeric properties. As a longer-term goal, catalyst recyclability represents a key challenge.

## Data accessibility

As a review article, there are no original data included.
